# Mental Health in Patients With Adrenal Incidentalomas: Is There a Relation With Different Degrees of Cortisol Secretion?

**DOI:** 10.1210/clinem/dgaa695

**Published:** 2020-10-05

**Authors:** Valentina Morelli, Alberto Ghielmetti, Alice Caldiroli, Silvia Grassi, Francesca Marzia Siri, Elisabetta Caletti, Francesco Mucci, Carmen Aresta, Elena Passeri, Flavia Pugliese, Annabella Di Giorgio, Sabrina Corbetta, Alfredo Scillitani, Maura Arosio, Massimiliano Buoli, Iacopo Chiodini

**Affiliations:** 1 Endocrinology Unit, Fondazione IRCCS Cà Granda Ospedale Maggiore Policlinico, Milan, Italy; 2 Department of Clinical Sciences and Community Health, University of Milan, Milan, Italy; 3 Department of Neurosciences and Mental Health, Fondazione IRCCS Ca’Granda Ospedale Maggiore Policlinico, Milan, Italy; 4 Unit for Bone Metabolism Diseases and Diabetes, Istituto Auxologico Italiano, IRCCS, Milan, Italy; 5 Endocrinology and Diabetology Service, IRCCS Istituto Ortopedico Galeazzi, Milan, Italy; 6 Unit of Endocrinology, Fondazione IRCCS Casa Sollievo della Sofferenza, San Giovanni Rotondo, Foggia, Italy; 7 Liaison Psychiatric Service, Unit of Neurology, Department of Emergency and Critical Areas, Fondazione IRCCS Casa Sollievo della Sofferenza, San Giovanni Rotondo, Foggia, Italy; 8 Department of Biomedical, Surgical and Dental Sciences, University of Milan, Milan, Italy; 9 Department of Pathophysiology and Transplantation, University of Milan, Milan, Italy; 10 Department of Medical Biotechnology and Translational Medicine, University of Milan, Milan, Italy

**Keywords:** subclinical hypercortisolism, adrenal incidentaloma, mental health, cognition

## Abstract

**Context:**

Cushing’s syndrome frequently causes mental health impairment. Data in patients with adrenal incidentaloma (AI) are lacking.

**Objective:**

We aimed to evaluate psychiatric and neurocognitive functions in AI patients, in relation to the presence of subclinical hypercortisolism (SH), and the effect of adrenalectomy on mental health.

**Design:**

We enrolled 62 AI patients (64.8 ± 8.9 years) referred to our centers. Subclinical hypercortisolism was diagnosed when cortisol after 1mg-dexamethasone suppression test was >50 nmol/L, in the absence of signs of overt hypercortisolism, in 43 patients (SH+).

**Interventions:**

The structured clinical interview for the *Diagnostic and Statistical Manual of Mental Disorders-5*, and 5 psychiatric scales were performed. The Brief Assessment of Cognition in Schizophrenia (Verbal and Working Memory, Token and Symbol Task, Verbal Fluency, Tower of London) was explored in 26 patients (≤65 years).

**Results:**

The prevalence of psychiatric disorders was 27.4% (SH+ 30.2% vs SH- 21.1%, *P* = 0.45). SH+ showed a higher prevalence of middle insomnia (by the Hamilton Depression Rating Scale) compared with SH- (51% vs 22%, *P* = 0.039). Considering the Sheehan Disability Scale, SH+ showed a higher disability score (7 vs 3, *P* = 0.019), higher perceived stress (4.2 ± 1.9 vs 2.9 ± 1.9, *P* = 0.015), and lower perceived social support (75 vs 80, *P* = 0.036) than SH-. High perceived stress was independently associated with SH (odds ratio [OR] = 5.46, confidence interval 95% 1.4–21.8, *P* = 0.016). Interestingly, SH+ performed better in verbal fluency (49.5 ± 38.9 vs 38.9 ± 9.0, *P* = 0.012), symbol coding (54.1 ± 6.7 vs 42.3 ± 15.5, *P* = 0.013), and Tower of London (15.1 vs 10.9, *P* = 0.009) than SH-. In 8 operated SH+, no significant changes were found.

**Conclusions:**

Subclinical hypercortisolism may influence patients’ mental health and cognitive performances, requiring an integrated treatment.

Glucocorticoids, mainly cortisol, play a crucial role in the allostatic process of adjustment to stressors and can determine important changes in central nervous system structures (1–3). It is well known that overt endogenous hypercortisolism is associated with psychiatric and neurocognitive impairment in about two-thirds of cases. The most important psychiatric disorders observed in Cushing’s syndrome (CS) are major depression, including the disturbance of appetite or sleep and mania and anxiety. Concerning cognitive functions, in CS the most frequent reported alterations are memory impairment (about 83% of cases) and reduced concentration (66% of cases) (4). Unfortunately, these alterations are only partially reversible after the hypercortisolism resolution (5, 6).

Although clinically silent, at least 20% of patients with incidentally found adrenal adenomas (adrenal incidentaloma, AI) may present with a mild hypercortisolism, less severe than CS, formerly called subclinical hypercortisolism (SH) or autonomous cortisol secretion (7). Though cardiovascular and bone consequences of these clinically silent adrenal masses have been largely explored and documented, data related to the impact of SH on mental health are scarce and, as regards to cognitive function, absent (8, 9).

In the present study we firstly aimed to explore mental health and cognitive functions in AI patients in relation to the presence of SH and, secondly, in a group of SH+ patients, the effect of adrenalectomy on mental health.

## Patients

**Figure 1.**
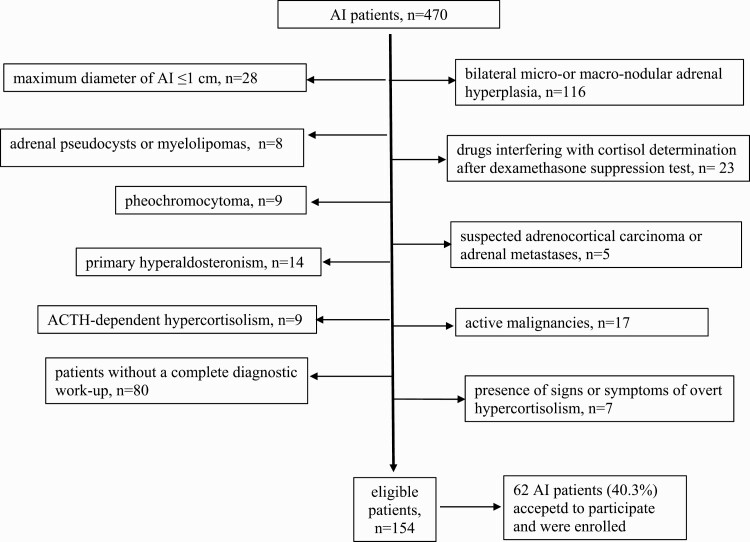
Enrolled patients after the application of exclusion criteria.

In this Italian multicenter study, we prospectively evaluated 470 consecutive patients with AI referred to the Endocrinology Unit of Fondazione Istituto di Ricovero e Cura a Carattere Scientifico (IRCCS) Ca’ Granda Ospedale Maggiore Policlinico, the Endocrinology and Diabetology Service of IRCCS Istituto Ortopedico Galeazzi in Milan, and the Endocrinology Unit of Fondazione IRCCS Casa Sollievo della Sofferenza in San Giovanni Rotondo, Foggia, from April 2016 to May 2019. The following exclusion criteria were applied: active malignancies, bilateral micro- or macronodular adrenal hyperplasia, maximum diameter of AI ≤1 cm, primary hyperaldosteronism, pheochromocytoma, suspected adrenocortical carcinoma or adrenal metastases, adrenal pseudocysts or myelolipomas, AdrenoCorticoTropic Hormone (ACTH)-dependent hypercortisolism, presence of signs or symptoms of overt hypercortisolism, steroid or gonadal therapy, or other drugs interfering with cortisol determination after a dexamethasone suppression test. Patients satisfying the inclusion criteria (n = 154) were asked to take part to the study: 62 (40.3%) patients accepted and were enrolled (Fig. 1); among these, 28 were also enrolled in an interventional, randomized trial supported by the Italian Ministry of Health (RF 2013-02356606 grant). Written, informed consent was obtained from all subjects, and the local Ethics Committees approved the study. Patients were divided in 2 groups: patients with subclinical hypercortisolism (SH+), if in the presence of serum cortisol after 1 mg dexamethasone overnight suppression test (1 mg DST) >50 nmol/L (ie, with at least possible autonomous cortisol secretion according to the European Society of Endocrinology-European Network for the Study of Adrenal Tumors [ESE-ENSAT] guidelines), and patients without SH (SH-) in the presence of serum cortisol levels after 1 mg DST ≤50 nmol/L (10). In a subanalysis, we performed a comparison of some cognitive variables among 3 groups (1–3), divided according to cortisol levels after 1 mg DST (<50, 50–138 and >138 nmol/L, respectively).

## Methods

All patients were evaluated by measuring: (1) plasma ACTH levels at 8:00 am (Immulite 2000, Siemens Medical Solutions Diagnostics, Los Angeles, California); (2) 24-hours urinary free cortisol (UFC) by liquid chromatography-mass spectrometry (LC–MS/MS), as previously described (11); (3) serum cortisol levels after 1 mg DST (Roche II, Elecsys Cortisol immunoassay, Roche Diagnostics, Mannheim, Germany [on CobaS E 602]) in at least 2 occasions. The latest available measurement was reported in the statistical analysis. In all patients, height and weight were measured and body mass index (BMI) calculated.

Data regarding diameter of the AI in the latest performed computed tomography scan and assumption of psychotropic drugs were also collected.

### Psychiatric evaluation

Trained psychiatrists, blinded to the hormonal profile, assessed all patients. Included subjects were first screened by the Structured Clinical Interview (SCID-5) for the *Diagnostic and Statistical Manual of Mental Disorders-5* (DSM-5), a semi-structured interview for the DSM-5 psychiatric diagnoses (12). The Global Severity of psychiatric symptoms were then assessed by the Clinical Global Impression (CGI) Severity of Illness scale (13), patients’ functioning by Global Assessement of Functioning (GAF) scale (14), severity of main psychiatric symptoms by the Brief Psychiatric Rating Scale (BPRS) (15), severity of depressive symptoms by the Hamilton Depression Rating Scale (HAM-D) (16), and severity of manic symptoms by the Young Mania Rating Scale (YMRS) (17). Psychiatrists evaluated sleep disturbances by the sleep diminution item of the YMRS scale (YMRSi) and initial (HAM1i), middle (HAM2i), and late insomnia (HAM3i) items of the HAM-D scale. Unfortunately, scores of these latter items were unavailable for 5 patients. We considered the subjects as being affected by sleep disorders in the case of HAM1i, HAM2i, HAM3i, or YMRSi scores ≥1 (16, 17). In addition, the Sheehan Disability Scale (SDS) (18) was used to measure the severity of dysfunction in work, social life/leisure activities, family life/home responsibilities (SDS-Disability), as well as the perceived levels of stress (SDS-Stress) and social support (defined as the percentage of the social support thought necessary for an adequately functioning SDS-Social support).

Eight out of 28 patients who were enrolled for the interventional, randomized trial, were re-evaluated after surgery in terms of mental health, 3 months after the interruption of steroid replacement therapy.

### Cognitive evaluation

Cognitive evaluation could be assessed in only 26 patients, aged ≤65 years. The choice to test cognition only in patients younger than 65 years was based on the high frequency of cognitive decline (eg, mild cognitive impairment) in elderly subjects independently from the presence of SH. Included subjects were evaluated by a clinical psychologist by the Italian version of Brief Assessment Cognition in Schizophrenia (BACS). Brief Assessment Cognition in Schizophrenia is a reliable tool to assess cognition in healthy subjects and in patients affected by psychiatric disorders (19–21). This battery includes tests to assess verbal memory (by a list learning task), working memory (by a digit sequencing task), motor speed (by a token motor task), verbal fluency (by a category instances and controlled oral words association test), attention and speed information processing (by a symbol coding task), and executive functions (by a Tower of London task). Data were expressed as corrected scores, adjusted for age, gender, and education where relevant, according to the multiple regression model used in the validation study of the Italian version of BACS (20).

### Statistical analysis

We performed statistical analysis by using SPSS version 25.0 statistical package software (SPSS Inc, Chicago, Illinois). For each variable the normality of distribution was tested by a Shapiro-Wilk test. Quantitative variables were expressed as mean ± standard deviation (SD) and range or median and interquartile range when not normally distributed. The SH+ and SH- groups were compared by independent sample *t*-tests or Mann-Whitney U tests, as appropriate. Qualitative variables were expressed as an absolute number count (percentage) and compared with chi-squared tests or Fisher’s exact tests, as appropriate. Bivariate associations among variables were tested by Pearson product moment (r) or Spearman’s rank correlation (r_s_), as appropriate. A logistic regression assessed the association between high levels of SDS-Disability, high levels of SDS-Stress, low levels of SDS-Social support (dependent variables), and the SH presence and possible confounding factors when appropriate (age, gender, and BMI). The comparison of quantitative variables among the 3 groups (1–3), divided according to cortisol levels after 1 mg DST (<50, 50–138 and >138 nmol/L, respectively) was performed by 1-way analysis of variance (ANOVA). Data of 8 SH patients before surgery and 3 months after stopping steroid replacement therapy were compared by paired sample *t*-tests. Two-tailed *P*-values <0.05 were considered statistically significant.

## Results

Clinical and hormonal variables of the whole cohort of the SH+ and SH- group are shown in Table 1; 64.5% of the patients were female and mainly distributed in the SH+ group. The 2 groups were comparable for age, BMI, and consumption of psychotropic drugs, and females were prevalent in the SH+ group. All female patients, except for 3 who reported regular menses, were in menopause. Among the male patients, none complained of symptoms or presented signs related to hypogonadism. As expected, in the SH+ group, we found lower plasma ACTH levels, higher cortisol levels after 1 mg DST, and a larger diameter of the adenoma when compared with the SH- group, though the UFC levels were similar in both groups. In 5 subjects, the UFC concentrations were above the upper limit of normal reference range, but in the absence of the clinical stigmata of CS.

**Table 1.** Clinical and hormonal variables in the whole sample of patients and divided into SH+ and SH- groups

Variables	All Patients (n = 62)	SH+ Group (n = 43)	SH- Group (n = 19)	*P*-value
Age, y	64.8 ± 8.9	64.5 ± 9.4	65.3 ± 7.8	NS
mean ± SD (range)	(30–79)	(30–76)	(51–79)	
Women, n (%)	40 (64.5)	32 (74.4)	8 (42.1)	0.014
Patients taking psychopharmacotherapy, n (%)	13 (21.0)	10 (23.3)	3 (15.8)	NS
BMI, kg/m^2^	26.7 ± 4.6	26.6 ± 4.7	27.1 ± 4.4	NS
mean ± SD (range)	(18.5–35.4)	(18.5–35.2)	(20.7–35.4)	
Diameter of AI, cm	2.8 ± 0.9	3.0 ± 0.9	2.4 ± 0.9	0.018
mean ± SD (range)	(1.1–5.3)	(1.5–5.3)	(1.1–5.0)	
ACTH (pmol/L)	2.3	1.9	4.2	<0.001
Median (interquartile range)	(2)	(1.1)	(1.9)	
1 mg DST (nmol/L)	66.2	88.3	34.2	<0.001
Median (interquartile range)	(64.3)	(55.2)	(13.8)	
UFC (nmol/24 h)	51.4	50.8	52.8	NS
Median (interquartile range)	(46.6)	(48.6)	(42.2)	
UFC > ULN, n (%)	5 (8.1)	4 (9.3)	1 (5.6)	NS

Data are expressed as mean values ± SD (range) or median (interquartile range) or absolute numbers, n and percentage in brackets (%). *P*-values referred to the comparisons between SH+ and SH- groups, *P*-values <0.05 were considered statistically significant; 1 mg DST, serum cortisol after 1 mg dexamethasone overnight suppression test; UFC > ULN, UFC > upper.

Abbreviations: ACTH, AdrenoCorticoTropic Hormone; AI, adrenal incidentaloma; BMI, body mass index; h, hours; NS, non-significant *P*-value; SD, standard deviation; SH, subclinical hypercortisolism; UFC, urinary free cortisol (24-hour); ULN, upper limit of normal; y, years.

### Psychiatric interview (SCID-5)

In 17 out of 62 patients (27.4%) a psychiatric disorder was diagnosed by the SCID-5 interview: 13 SH+ and 4 SH- (30.2% vs 21.1% in the SH+ and SH- groups, respectively; *P* = 0.45). Specifically, 11 patients were diagnosed with generalized anxiety disorder (GAD): 9 from the SH+ group and 2 from the SH- group (21% vs 10.5%, respectively; *P* = 0.32). Among the non-GAD SH+ patients, 2 patients were diagnosed with bipolar disorder (versus 0 in the SH- group), 1 patient with cyclothymic disorder, and 1 patient with unspecified depressive disorder. Among the non-GAD SH- patients, we observed 1 patient with a major depressive disorder and 1 patient with a panic disorder. Differently from SH+ group, in the SH- group, patients diagnosed with a psychiatric disorder showed higher UFC levels (*P* = 0.025) compared with SH- patients without a psychiatric condition, while no differences in ACTH levels or cortisol after 1 mg DST were observed (data not shown). Among the 5 patients with UFC > upper limit of normal (ULN), 2 were diagnosed with GAD (1 belonging to the SH+ group and 1 belonging to the SH- group).

### Psychiatric rating scales

The median scores derived from the psychiatric HAM-D, BPRS, YMRS, and CGI scales did not significantly differ between the SH+ and SH- groups. With regard to the SDS item mean total scores, SH+ patients were found to have higher levels of disability related to mental illness (SDS-Disability), higher levels of perceived stress (SDS-Stress), and lower levels of perceived social support (SDS-Social support) as compared with SH- patients (Table 2). The logistic regression analysis showed that high levels of SDS-Disability (≥4 in a scale of 30) were significantly associated with the SH+ condition (odds ratio [OR] = 5.5, CI 95% 1.5–20.4, *P* = 0.01) regardless of age (OR = 0.97, CI 95% 0.9–1.0, *P* = 0.493), gender (OR = 1.39, CI 95% 0.4–4.9, *P* = 0.606) and BMI (OR = 1.0, CI 95% 0.9–1.1, *P* = 0.926). Similarly, high levels of SDS-Stress (≥4 in a scale of 10) were significantly associated with the SH + condition (OR = 5.9, CI 95% 1.4–22.8, *P* = 0.015), but not with female gender (OR = 1.6, CI 95% 0.5–5.8, *P* = 0.44), BMI (OR = 1.09, CI 95% 1–1.2, *P* = 0.18) nor with age (OR = 0.97, CI 95% 0.9–1.0, *P* = 0.51). Moreover, low levels of SDS-Social support (<70 in a scale of 100) were associated with the presence of SH + condition (OR = 3.7, confidence interval (CI) 95%: 1.0–14, *P* = 0.05) but not with age (OR = 1.0, CI 95%: 0.9–1.0, *P* = 0.744) nor with gender (OR = 1.8, CI 95%: 0.5–6.3, *P* = 0.345). Considering hormonal and psychiatric variables, UFC levels showed a direct correlation with BPRS total scores (rs = 0.35, *P* = 0.005) and ACTH levels were inversely correlated with SDS-disability scores (rs = -0.28, *P* = 0.029). Analyzing specific items included in HAM-D and YMRS sleep scales, we observed a higher frequency of middle insomnia (HAM2i) in SH+ compared with SH- patients (Table 3). There was also a trend towards an increased prevalence of sleep diminution (YMRSi) in the SH+ group compared with the SH- group (*P* = 0.1). Considering hormonal and psychiatric variables, UFC levels showed a weak direct correlation with the scores of sleep reduction: YMRSi (rs = 0.27, *P* = 0.042).

**Table 2.** Psychiatric rating scale scores and the percentage of subjects with clinically significant psychiatric symptoms in SH+ and SH- groups

	SH+ (n = 43)	SH- (n = 19)	*P*-value
Depression—HAM-D (0–34)	5	5	NS
Median (interquartile range)	(3)	(4)	
*Clinically significant HAM-D (>7), n (%)*	*9 (20.9)*	*3 (15.8)*	NS
BPRS (0–126)	23	23	NS
Median (interquartile range)	(5)	(4)	
*Clinically significant BPRS (≥31), n (%)*	*1 (2.3)*	*0 (0)*	NS
Mania—YMRS (0–36)	4	4	NS
Median (interquartile range)	(3)	(6)	
*Clinically significant YMRS (≥10), n (%)*	*3* (7)	*0 (0)*	NS
Impression of severity—CGI (1–7)	1	1	NS
Median (interquartile range)	(1)	(0)	
*Clinically significant CGI (>1) n (%)*	*16 (37.2)*	*4 (21.1)*	NS
Functioning—GAF (0–100)	82.7 ± 6.1	82.5 ± 7.9	NS
Mean ± SD (range)	(68–92)	(63–94)	
SDS—Disability (0–30)	7	3	0.019
Median (interquartile range)	(7)	(6)	
SDS—Stress (0–10)	4.2 ± 1.9	2.9 ± 1.9	0.015
Mean ± SD (range)	(0–8)	(0–7)	
SDS—Social Support (0–100)	75	80	0.036
Median (interquartile range)	(40)	(30)	

In the left column for each scale, theoretical extremes, if available, are reported in brackets. Quantitative variables are expressed as mean ± SD (range) or median (interquartile range) when non-normally distributed. For HAMD, BPRS, YMRS, and CGI-S scales, the number of patients with scores beyond the threshold conventionally considered as clinically relevant and the percentage (in brackets) are also reported in italics. *P*-values <0.05 were considered statistically significant, NS: not significant P value.

Abbreviation: BPRS, Brief Psychiatric Rating Scale; CGI, Clinical Global Impression; GAF, Global Assessement of Functioning; HAM-D, Hamilton Depression Rating Scale; NS, non-significant *P*-value; SD, standard deviation; SDS, Sheehan Disability Scale; SH, subclinical hypercortisolism; YMRS, Young Mania Rating Scale.

**Table 3.** Patients with sleep disorders

	SH+ (n = 39)	SH- (n = 18)	*P*-value
*Presence of HAM1i (%)*	10 (25.6)	6 (33.3)	NS
*Presence of HAM2i (%)*	20 (51.3)	4 (22.2)	0.039
*Presence of HAM3i (%)*	12 (30.8)	4 (22.2)	NS
*Presence of YMRSi (%)*	22 (56.4)	6 (33.3)	NS

YMRSi is the sleep diminution item of the YMRS scale. We considered the presence of HAM1i, HAM2i, HAM3i, and YMRSi for scores ≥1 for each item. Data are expressed as absolute numbers and percentages in brackets.

Abbreviations: HAM1i, HAM2i, HAM3i, initial, central and late insomnia evaluated by Hamilton Depression Rating Scale; HAM-D, Hamilton Depression Rating Scale; SH, subclinical hypercortisolism; YMRS, Young Mania Rating Scale.

### Cognitive evaluation (BACS)

The BACS cognitive evaluation was conducted only in patients aged ≤65 years. We did not observe any statistically significant difference between SH+ and SH- patients regarding verbal memory as well as working memory, neither in absolute numbers nor as percentage of cases with impaired cognition. Interestingly, SH + patients performed better in verbal fluency, symbol coding, and Tower of London tasks as absolute scores than SH- ones. Moreover, with regard to symbol coding and Tower of London tasks, we observed a higher frequency of impaired cognition in SH- subjects than in SH+ ones (Table 4).

**Table 4.** Cognitive evaluation by BACS in SH+ and SH- patients

	SH+ (n = 16)	SH- (n = 10)	*P*-value
**Verbal memory**	48.8 ± 10.7	52.0 ± 7.8	NS
Mean ± SD (range)	(21.5–65.5)	(38.5–64.3)	–
*Impaired verbal memory (<33), n (%)*	1 (6.3)	0 (0)	NS
**Working memory**	21.8	23.9	NS
Median (interquartile range)	(8.6)	(18.4)	–
*Impaired working memory (<14.9), n (%)*	3 (18.8)	1 (10)	NS
**Token task**	67.7 ± 8.6	70.6 ± 10.3	NS
Mean ± SD (range)	(49.5–83.5)	(54.75–85.7)	–
*Impaired token task (<68.7), n (%)*	11 (68.8)	5 (50)	NS
**Verbal fluency**	49.5 ± 38.9	38.92 ± 9	0.012
Mean ± SD (range)	(31.5–72.2)	(26.75–58.5)	–
*Impaired verbal fluency (<31.6), n (%)*	1 (6.3)	2 (20)	NS
**Symbol coding**	54.1 ± 6.7	42.27 ± 15.5	0.013
Mean ± SD (range)	(38.5–63.3)	(7.75–59.5)	–
*Impaired symbol coding (<40.5), n (%)*	1 (6.3)	4 (40)	0.055
**Tower of London**	15.1	10.9	0.009
Median (interquartile range)	(4.1)	(3.6)	–
*Impaired Tower of London (<12.4), n (%)*	2 (12.5)	6 (60)	0.026

Quantitative variables are expressed as the mean ± SD (range) or median (interquartile range) if non-normally distributed. The number of cases with impaired performance and percentages in brackets are also reported for each task. The cutoffs for cognitive deficits are defined in the validation study of the Italian version of the BACS (20).

Abbreviations: BACS, Brief Assessment Cognition in Schizophrenia; NS, not statistically significant; SD, standard deviation.

**Figure 2.**
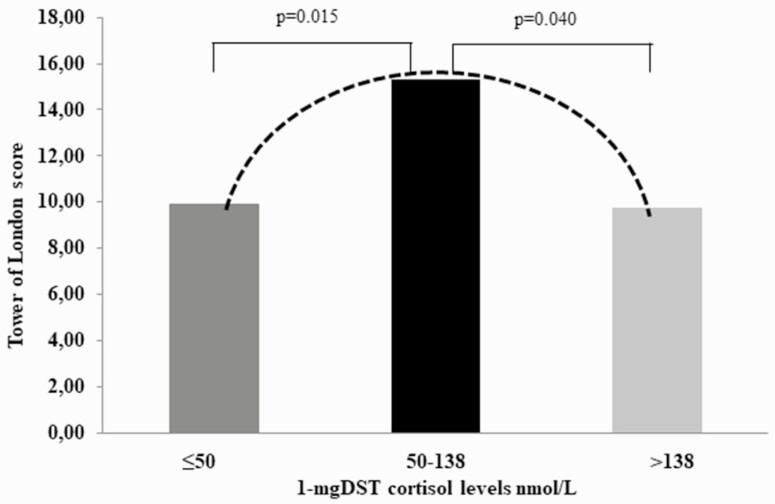
Tower of London task mean scores in 3 groups of patients according to cortisol levels after 1 mg DST: patients with cortisol ≤50 nmol/L (dark grey); patients with cortisol between 50 and 138 nmol/L (black); and patients with cortisol >138 nmol/L (light grey).

Among cognitive variables, verbal fluency was positively correlated with symbol coding (r_s_ = 0.78, *P* < 0.001) and Tower of London tasks (rs = 0.39, *P* = 0.049). With regard to functional parameters, UFC levels were directly correlated with performances in symbol coding task (rs = 0.50, *P* = 0.009), but inversely correlated with the verbal memory ones (rs = -0.43, *P* = 0.027); cortisol levels after 1 mg DST were directly correlated with performances in the Tower of London task (rs = 0.40, *P* = 0.043). Moreover, the diameter of AI was directly correlated with performances in verbal fluency (rs = 0.58, *P* = 0.002) and symbol coding tasks (rs = 0.67, *P* < 0.001). To better explore this association we further divided SH+ patients into 2 groups according to cortisol levels after 1 mg DST, thus obtaining 3 groups of patients: those with cortisol ≤50 nmol/L (group 1 corresponding to SH-, without autonomous cortisol hypersecretion, n = 10), those with cortisol between 50 and 138 nmol/L (group 2, with possible autonomous cortisol secretion, n = 13), and those with cortisol >138 nmol/L (group 3, with autonomous cortisol hypersecretion, n = 3) (10). With regard to the Tower of London task mean scores, we found that patients of group 2 performed better than patients in both groups 1 and 3 (Fig. 2).

### Surgical outcome

All the SH+ patients who underwent adrenalectomy (n = 8) received glucocorticoid replacement therapy for at least 1 month after surgery. Then, in the presence of adrenal insufficiency, hypothalamic–pituitary–adrenal (HPA) axis function was re-evaluated every 3 months by Synacthen test. The mean duration of glucocorticoid replacement therapy was 9.3 ± 6.2 months (range 1–19). Parameters of adrenal function and psychiatric evaluations were reassessed 3 months after the interruption of steroid replacement therapy.

Comparing basal with postsurgical parameters, we observed a significant increase in mean ACTH levels (1.9 ± 0.5 pmol/L vs 6.3 ± 4.2 pmol/L, *P* = 0.02, respectively), a reduction of cortisol levels after 1 mg DST (91.1 ± 35.9 nmol/L vs 27.0 ± 11.3 nmol/L, *P* = 0.017, respectively), and a reduction of UFC levels, even though without reaching statistical significance (78.1 ± 71.2 nmol per 24 hours vs 37.5 ± 26.9 nmol per 24 hours, *P* = 0.227). The 2 patients with a psychiatric diagnosis at baseline (1 with cyclothymia and 1 with GAD) maintained their diagnosis after surgery. Overall, we did not find any significant improvement in any psychiatric scores. However, the SDS-Disability score tended to improve, but without achieving statistical significance (8.1 ± 4.6 vs 4.9 ± 4.6, *P* = 0.140, before and after surgery, respectively); similarly, after surgery, we found a negative correlation between variation of ACTH levels and variation of SDS-Disability levels (r = -0.60, *P* = 0.12), although without reaching statistical significance.

## Discussion

This is the first study that explores, in AI patients, the effects of clinically silent hypercortisolism and adrenalectomy on mental health by the use of a comprehensive battery of psychiatric rating scales and cognitive tests.

Our results did not show a higher prevalence of psychiatric disorders in SH+ patients compared with SH- ones. However, in the SH+ group we observed increased levels of disability related to mental illness, higher levels of perceived stress, lower levels of perceived social support, and a higher prevalence of middle insomnia than in the SH- group. Interestingly, SH+ patients showed better cognitive functions in specific items, namely verbal fluency, attention and information processing, and executive planning functions, than SH- patients. Finally, in the SH+ group, 3 patients showed bipolar spectrum disorders that are known to be associated with increased activity of the hypothalamus-pituitary-adrenal axis (22). Overall, these findings are of clinical interest since, in the general population, the prevalence of adrenal adenoma and SH are elevated, reaching the 4% to 9% and 0.2% to 2%, respectively (23). Therefore, our results confirm and reinforce the idea that even a low degree of cortisol excess may be deleterious for psychological health. Indeed, in the past years, a study performed by generic questionnaires suggested a reduction of health-related quality of life (QoL) in AI patients; however, the role of the degree of cortisol excess has not been elucidated (24). Subsequently, the possibility of a QoL improvement after adrenalectomy in SH patients has been reported (25), suggesting that the QoL impairment could be a further consequence of SH. The present results clearly show that patients with SH have a reduction of psychological health and QoL.

The possible causative role of a low degree of cortisol excess on psychiatric and neurocognitive impairment is explainable considering cortisol plays a crucial role in the allostatic process of adjustment to stressors and can determine important changes in central nervous system structures. Likewise, CS is associated with psychiatric and neurocognitive impairment in the majority of patients (2–4), with these alterations being only partially reversible with the hypercortisolism resolution (5, 6). One of the most important psychiatric disorders observed in CS is major depression, including the disturbance of appetite or sleep (4). Although we did not find a high prevalence of major depression in the SH+ group, these patients presented with a higher prevalence of middle insomnia, resembling the feature detected in CS patients (26, 27). Sleep reduction has a pivotal role in determining the QoL and increasing data show that poor quality of sleep may increase the risk of cardiovascular diseases (28). We speculate that sleep disturbances may be caused by even mild degree of hypercortisolism, while major depression is mainly related to overt cortisol excess. Of note, sleep disturbances often represent the first sign of the onset of a depressive episode and in any case, they represent a risk factor for future of depression (29). In support of this hypothesis, the occurrence of major depression in CS is mainly related to elevated UFC levels, which are generally in the normal range in patients with SH (30). The prevalence of anxiety disorders, detected in 21% of SH+ patients, is higher than the 5% reported in the general population. The anxiety disorders’ prevalence remains higher than expected, although in our cohort of SH+ patients we have a large representation of female subjects, in whom the risk of GAD is doubled (31). Interestingly, we found 3 GAD cases and 1 major depression case among SH- patients, characterized by UFC levels higher than those observed in patients without psychiatric disorders, further suggesting the close relationship between psychiatric health and the degree of cortisol secretion, even in patients considered without SH. This is in agreement with recent data suggesting that some chronic metabolic disorders, such as hypertension, diabetes, and osteoporosis may be related to cortisol secretion, sensitivity, and peripheral activity even in subjects without adrenal adenoma or hypercortisolism (32, 33).

The evidence of an impact of the SH condition on QoL in the present study is furthermore confirmed by the finding that SH+ patients had higher levels of SDS-Disability, higher levels of SDS-Stress, and lower levels of SDS-Social support as compared with SH- patients. In particular, perceived stress, which could be expected to be higher in women, was independent of gender, age, and BMI, possible confounding factors. Similarly, low levels of SDS-Social support were associated with SH regardless of age. Unfortunately, we do not have data about socioeconomic status. However, the relation between socioeconomic status and psychological health can be considered bidirectional. Indeed, a low socioeconomic status could be considered the cause as well as the result of a psychiatric disturbance. Even though we cannot provide a clear-cut idea on the surgical outcome of these patients, in the light of the small sample size and the short follow-up, the finding that the SDS-Disability score slightly improves after surgery suggests a reversibility of the perceived stress in SH patients. However, in the absence of a control group and a longer follow-up, it is not possible to exclude that, as in CS patients, the impairment of QoL could be a lifelong persistence (5).

Differently from most CS patients experiencing memory impairment and reduced concentration (4), these alterations were not found in SH+ patients. Nonetheless, SH+ patients performed better in verbal fluency, information processing speed (symbol coding task), and executive planning functions (Tower of London task) than SH- patients. The differences detected between SH+ and SH- patients could be explained if we consider that symbol coding, verbal fluency, and Tower of London require the activation of the prefrontal cortex (34–36) whose performance is demonstrated to be improved by a little amount of exogenous steroids (37). The finding that, in the Tower of London task, patients with an intermediate degree of cortisol secretion (ie, those with cortisol after 1 mg DST between 50 and 138 nmol/L) performed better than patients with a higher degree of cortisol secretion (ie, those with cortisol after 1 mg DST above 138 nmol/L) and without SH (Fig. 2) is in keeping with the idea that a little amount of cortisol hypersecretion may improve these cognitive functions. The association of the degree of cortisol secretion with features of cognitive functions mimics the inverted U adaptive stress response (2), in which intermediate cortisol levels are associated to a positive hyperactivity of central nervous system, while a further increase of cortisol levels are associated with a maladaptive response. To confirm this hypothesis and to elucidate the reasons why our findings in cognitive tasks were quite the opposite of those described in CS, we would need a larger sample of patients, possibly including subjects with overt hypercortisolism.

We are aware that the present study suffers from some limitations. Firstly, the sample size of patient who underwent cognitive function evaluation was limited. However, we are confident that our results are informative, as this item has never been evaluated so far, and, importantly, at variance with previous studies (24, 38), our cases were diagnosed by a psychiatrist and not by generic self-administered QoL questionnaires, thus increasing the diagnostic accuracy. Secondly, we have a higher prevalence of SH+ (69%) than SH- patients, probably due to a greater concern of SH+ patients’ health. This apparently high prevalence of SH+ patients is in agreement with the use, for the SH diagnosis, of a cutoff for cortisol after 1 mg DST set at 50 nmol/L. Indeed, by using this cutoff, the prevalence of this condition may exceed 40% of AI patients (39). Moreover, we recognize that a recruiter bias might be present, as well: between 2016 and 2019, due to the randomized trial supported by Italian Ministry of Health (RF 2013-02356606 grant) specifically designed to evaluate the effects of surgery in SH+ patients, conducted in the 3 centers involved in this study, a high number of SH+ patients, larger than usual, has been evaluated, specifically sent by other centers. A third limitation of the study is the absence of a real control group of healthy subjects. Indeed, the inclusion of a control group of subjects without AI could have been more informative for the possibility that, even in AI patients without an SH diagnosis, the degree of cortisol secretion could in fact impact QoL, as suggested for other complications of cortisol excess, such as hypertension, diabetes, and osteoporosis (32, 33). Lastly, the lack of correlation between cortisol levels and different analyzed items suggests that cortisol secretion might not entirely explain the QoL impairment in SH. Therefore, data regarding individual sensitivity to glucocorticoids (eg, glucocorticoid [GC] receptor polymorphisms and 11β-hydroxysteroid dehydrogenase activity) or androgen levels could have been useful, considering the potential role of these specific factors in neuropsychiatric illness (40–42). Certainly, our study does not demonstrate causality but only associations. The only findings supportive of possible causality are the suppressed baseline ACTH levels that increase after surgery and the inverse correlation of ACTH levels with SDS disability scores. It is, however, important to notice that the presence of low ACTH levels permits to differentiate an SH sustained by an adrenal adenoma from a pseudo-CS, recently referred to as physiologic/non-neoplastic hypercortisolism (43). Indeed, pseudo-CS is characterized by an increase in HPA axis activity, and two meta-analyses showed higher ACTH levels in both bipolar and depressed individuals compared with healthy subjects (22, 44). Therefore, in our opinion, our exclusion criterion of ACTH-dependent hypercortisolism should have made it unlikely to have enrolled “pseudo-Cushing patients” in the SH+ group. Regarding the psychiatric features of “ACTH-independent subclinical hypercortisolism,” we could expect them to be rather similar to those of “pseudo-Cushing patients,” as no differences between pituitary-dependent and independent CS were described (45).

In conclusion, besides the metabolic complications, mental health should be evaluated in AI patients. Indeed, SH may be associated to increased levels of disability related to mental illness, higher levels of perceived stress, lower levels of perceived social support, and a higher prevalence of middle insomnia. On the other hand, the presence of slight cortisol excess may be associated with better performance in some cognitive functions. These data could be useful for ameliorating our protocols for the diagnostic work-up, addressing the treatment of choice and setting-up rehabilitative procedures in patients with AI.

## Data Availability

Some or all datasets generated during and/or analyzed during the current study are not publicly available but are available from the corresponding author on reasonable request.

## Additional Information

***Disclosure Summary:*** The authors have nothing to disclose.
